# Transcriptomics in Venous Leg Ulcers (VLU): A Systematic Review

**DOI:** 10.1111/wrr.70140

**Published:** 2026-03-05

**Authors:** Chien Lin Soh, Kia Hau Matthew Tan, Alun Huw Davies, Sarah Onida

**Affiliations:** ^1^ Section of Vascular Surgery, Department of Surgery and Cancer Imperial College London London UK

**Keywords:** RNA, transcriptomics, venous disease, venous insufficiency, venous ulcers

## Abstract

Venous leg ulcers (VLUs) are chronic wounds in the lower limbs that cause significant morbidity. The underlying biology underpinning non‐healing in VLUs is still poorly understood and differences in transcriptomic profiles may help elucidate biological pathways involved in wound chronicity. A systematic review was conducted in accordance with Preferred Reporting Items for Systematic Reviews and Meta‐Analyses. Studies were included if transcriptomes from adults with confirmed VLU were described. A search of EMBASE, OVID and PubMed between 1 January 1994 and 17 August 2024 yielded 429 articles; 34 studies were included after full‐text screening by two independent reviewers. Twenty‐nine studies investigated coding messenger RNA (mRNA) expression, while five studies investigated non‐coding RNA. Samples were obtained from wound biopsies (*n* = 31) or whole blood (*n* = 3). There were variations in mRNA expression across comparisons between patients with VLUs and controls such as tissue from other patients, tissue from different sites and treated tissues. Altered levels of mRNA transcripts suggest chronic inflammatory states, hyperproliferation and reduced wound healing. Studies exploring non‐coding RNAs described their roles in regulating wound repair. Paired analyses of microRNA expression demonstrate impact on RNA expression profiles, with effects on inhibition of wound healing. Transcriptomic analysis provides new insights into the pathophysiology of the development and healing of VLUs. Further development of RNA biomarkers and gene expression profiles can improve diagnosis and prognostication of VLUs, but heterogeneity of current data may make it difficult to draw clinical significance.

**Trial Registration:** PROSPERO: CRD42024580526

## 1 Background

Venous leg ulcers (VLUs) are a chronic condition affecting 1%–2% of the population that has an increasing prevalence with age [1]. VLUs are the most severe manifestation of chronic venous disease (CVD) and present a significant burden on patients and healthcare systems [2, 3]. These chronic wounds arise from impaired venous return and micro‐circulatory dysfunction, leading to tissue breakdown and long‐term disability and pain for patients. VLUs can be further complicated by infection and delayed healing, requiring judicious wound care and regular clinic visits. VLUs impact the physical and psychological quality of life of patients significantly, arising from restrictions in mobility and the presence of chronic pain [4, 5]. VLUs also result in a great cost to healthcare systems worldwide, with an estimated annual cost of over £2 billion in the United Kingdom (UK) spent on treating VLUs alone [6, 7].

The gold standard in VLU care is wound care, compression therapy and treatment of superficial venous reflux [8]. Additional measures that may have a positive prognostic impact on VLU healing are patient education and a healthy diet and exercise. Adjuvant therapies to support healing of VLUs include skin grafting, ultrasound technology, oxygen therapy, biologics and stem cell transplants—although these therapies are not common practice in the United Kingdom due to a limited clinical evidence base. Despite treatment, VLUs can take up to 12 months to heal [9]. The prognosis of a VLU is affected by a myriad of factors including the ulcer's size and age, and the presence of untreated venous disease [10]. Despite regular treatment, VLUs may fail to heal or may recur—a significant challenge in the development of appropriate wound care pathways [11, 12]. The ESCHAR and EVRA trials demonstrated that patients with VLUs undergoing compression therapy have around 60%–75% healing rates [13, 14, 15]. However, real world data suggest that healing rates outside of the context of clinical trials may be significantly lower [11].

VLUs arise from complex interactions between venous, lymphatic, genetic and environmental factors. These biological pathways are complex and poorly understood, with limited diagnostic and therapeutic options. There is a need to increase understanding of the basic biology behind VLU development and healing, and the study of transcriptomics can support this increased understanding.

Transcriptomics refers to the techniques employed to study RNA transcripts produced by the genome. Messenger RNA (mRNA) serves as an intermediary messenger during the process of transcription, where the genome is expressed into proteins. Transcriptomic studies provide data on the presence and quantity of mRNA transcripts from samples at a specific time‐point. A myriad of technologies including microarrays, high‐throughput sequencing (HTS) and next‐generation sequencing (NGS) has driven the data acquired on the transcriptome [4, 5, 6]. Further development in the analysis of non‐coding RNAs including long non‐coding RNA (lncRNAs), small non‐coding RNAs (sncRNAs), micro RNAs (miRNAs) and circular RNAs (circRNAs) have revealed their regulation of the transcriptome either through pre‐translational modifications or direct regulation of translation itself. Measurement of a snapshot of relevant RNAs in the tissues of VLUs may provide insight into how genes are expressed, regulated and transcribed in the context of VLUs.

This systematic review therefore aims to summarise the current literature on the transcriptome in patients with VLUs and the differential expression of the transcriptome in relation to changes in clinical parameters and treatments. This knowledge on transcriptomics can contribute to a holistic understanding of VLUs, supporting future research into the development of effective and safe therapies for VLUs.

## 2 Methods

A systematic review was conducted in accordance with Preferred Reporting Items for Systematic Reviews and Meta‐Analyses (PRISMA) [16, 17]. The systematic review was prospectively registered on PROSPERO (ID CRD42024580526) [18].

The search strategy was designed with the following inclusion criteria: transcriptomic studies analysing samples from human lower limb ulcers with confirmed venous origin using duplex ultrasound or relevant investigations. The search terms are described below (Table 1). A filter was applied to include English language studies.

**TABLE 1:** Search strategy used for PubMed, EMBASE and Ovid, accessed on 10 July 2025.

PubMed search strategy	
1	(“Varicose ulcer*” OR “leg ulcer*” OR “venous ulcer*” OR “VLU*” OR “venous wound*” OR “stasis ulcer*” OR “crural ulcer*” OR “ulcus cruris” OR “ulcer cruris”)
2	(Sequence Analysis, RNA [MeSH Terms] OR High‐Throughput RNA Sequencing [MeSH Terms] OR Gene Regulatory Networks/genetics* [MeSH Terms] OR Genomics/methods* [MeSH Terms] OR RNA/genetics* [MeSH Terms] OR Gene Expression Profiling [MeSH Terms] OR Transcriptome* [MeSH Terms] OR Transcriptome/genetics* [MeSH Terms] OR RNA‐seq*[Title/Abstract] OR “transcriptome profiling” [tiab] OR “transcriptome analysis” [tiab] OR “transcriptional dysregulation” [tiab] OR “genome‐wide expression profiling” [tiab] OR “genome‐wide expression analysis” [tiab] OR “differential* express*” [tiab] OR “mRNA‐seq” [tiab] OR “transcriptome sequencing” [tiab] OR “transcriptional signatures” [tiab] OR “transcriptional alterations” [tiab] OR “transcriptional changes” [tiab]) OR (‘transcrip*’ OR ‘transcriptome’ OR ‘gene expression’ OR ‘gene profile’ OR ‘RNA’) OR (‘sequenc*’ OR ‘sequencing’ OR ‘RNAseq’ OR ‘microarray’ OR ‘array’ OR ‘cDNA’ OR ‘AWymetrix’ OR ‘Agilent’) OR ((“messenger RNA” OR mRNA) OR (microRNA OR miRNA or miR) OR (“long non‐coding RNA” OR lncRNA) OR (“circular RNA” OR circRNA) OR (“transfer RNA” OR tRNA)) OR (“next generation sequencing” OR “third generation sequencing” OR “RNA microarray” OR “RNA sequencing” OR “RNA‐seq” OR “RNA seq”) OR (“genome expression” OR “genetic expression profiling”)
3	1 AND 2

Ovid search strategy	
1	(“Varicose ulcer*” or “leg ulcer*” or “venous ulcer*” or “VLU*” or “venous wound*” or “stasis ulcer*” or “crural ulcer*” or “ulcus cruris” or “ulcer cruris”).mp. [mp = tx, bt, ti, ab, ct, sh, hw, tn, ot, dm, mf, dv, kf, fx, dq, cw, nm, ox, px, rx, an, ui, ds, on, sy, ux, mx, tc, id, tm]
2	(“Transcrip*” or “Transcriptome*” or “transcriptome profiling” or “transcriptome analysis” or “transcriptional analysis” or “transcriptional dysregulation” or “genome‐ wide expression profiling” or “gene expression” or “genome expression” or “genetic expression profiling” or “Gene profile” or “genome‐wide expression analysis” or “differential* express*” or “mRNA‐seq” or “transcriptome sequencing” or “transcriptional signatures” or “transcriptional alterations” or “transcriptional changes” or (“messenger RNA” or mRNA or (microRNA or miRNA or miR) or (“long non‐coding RNA” or lncRNA) or (“circular RNA” or circRNA) or (“transfer RNA” or tRNA)) or (“next generation sequencing” or “third generation sequencing” or “RNA microarray” or “RNA sequencing” or “RNA‐seq” or “RNA seq”) or (Sequence Analysis RNA or High‐Throughput RNA Sequencing or Gene Regulatory Networks)).mp. [mp = tx, bt, ti, ab, ct, sh, hw, tn, ot, dm, mf, dv, kf, fx, dq, cw, nm, ox, px, rx, an, ui, ds, on, sy, ux, mx, tc, id, tm]
3	1 AND 2

Abstract and full‐text screening were completed by two independent reviewers (C.L.S. and K.H.M.T.), with any conflicts referred to a third senior reviewer (S.O.). Exclusion criteria included patients with arterial, diabetic or mixed ulcers, sickle cell ulcers, or animal models. Full‐text extraction that met inclusion criteria were analysed and metadata extracted for each study (C.L.S. and K.H.M.T.). Risk of bias assessment using the Cochrane ROBINS‐E tool for observational studies and ROB2 tool for randomised controlled trials was performed on included studies.

## 3 Results

**FIGURE 1:**
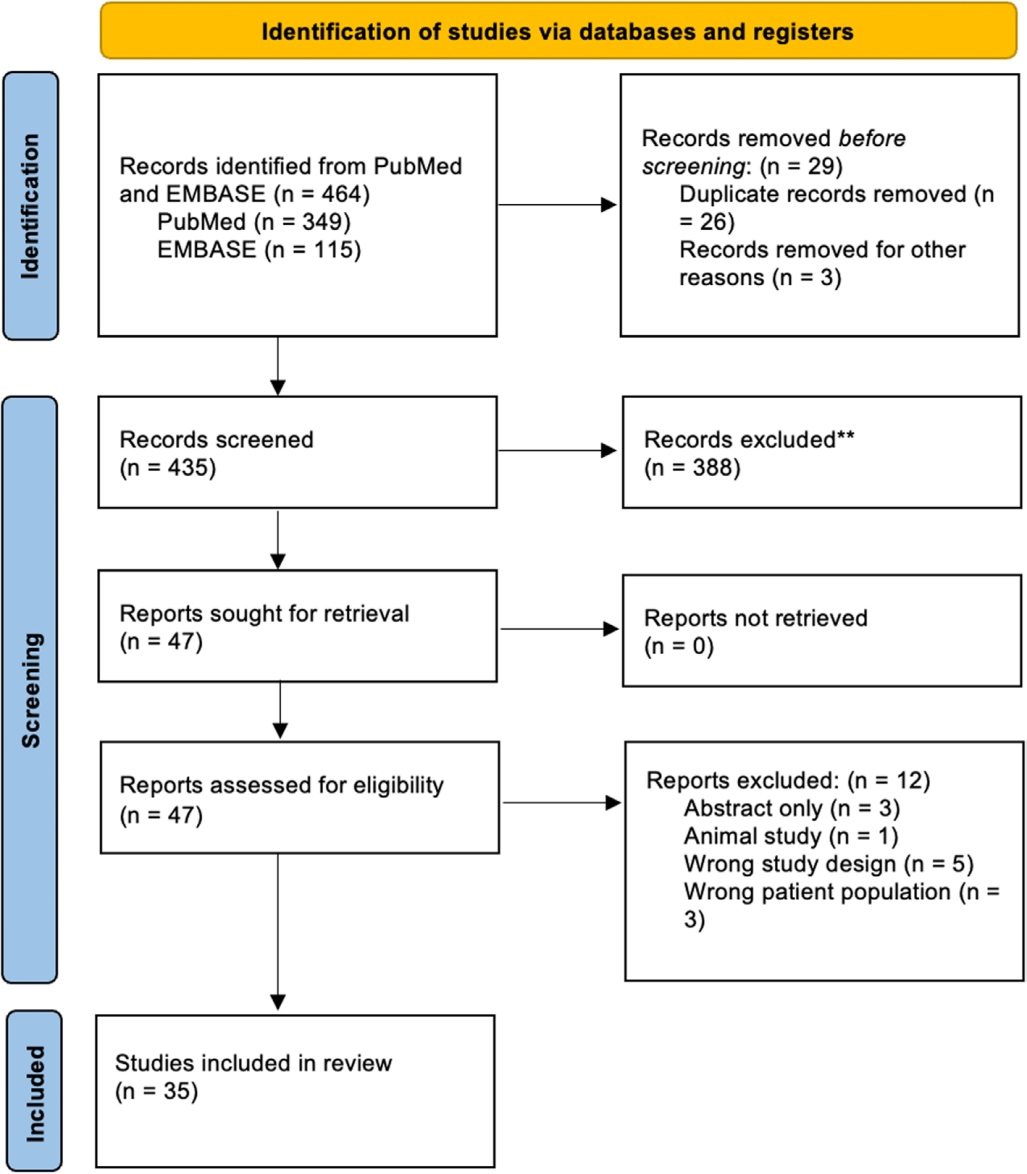
PRISMA flowchart indicating abstract and full‐text screening process for included study selection (*n* = 35). PRISMA 2020 flow diagram for new systematic reviews which included searches of databases and registers only.

A search of EMBASE, OVID and PubMed between 1 January 1994 and 10 July 2025 yielded 464 articles; 35 studies were included after full‐text screening by two independent reviewers (Figure 1) [16, 17]. Twenty‐nine studies investigated coding mRNA expression while five studies investigated non‐coding RNA. Samples were obtained from wound biopsies (*n* = 31) or blood human peripheral blood mononuclear cells (PBMC) (*n* = 3). There was a range of sample sizes across these studies, ranging from three to 71 samples.

**FIGURE 2:**
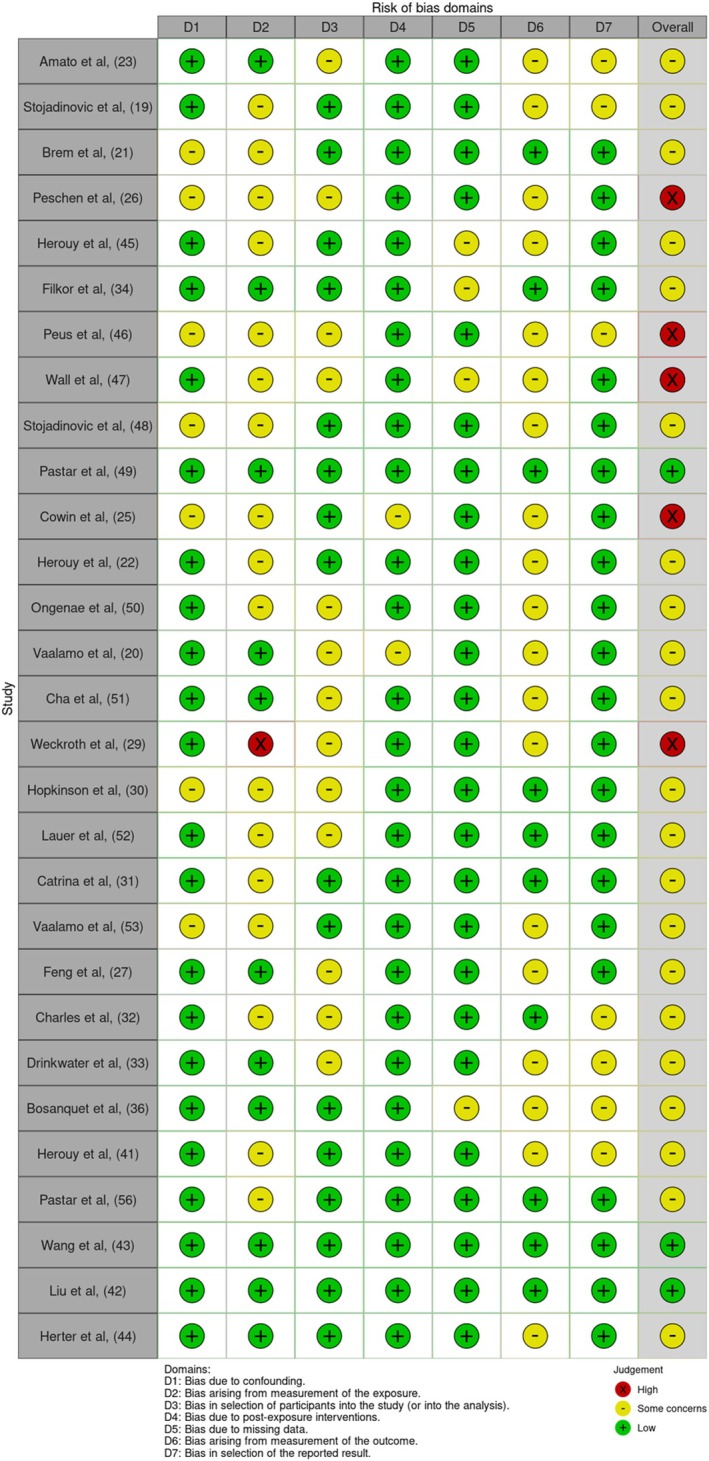
Risk of bias assessment visualised using the Risk‐of‐bias VISualisation (robvis) tool using ROBINS‐E framework (*n* = 30) [19].

**FIGURE 3:**
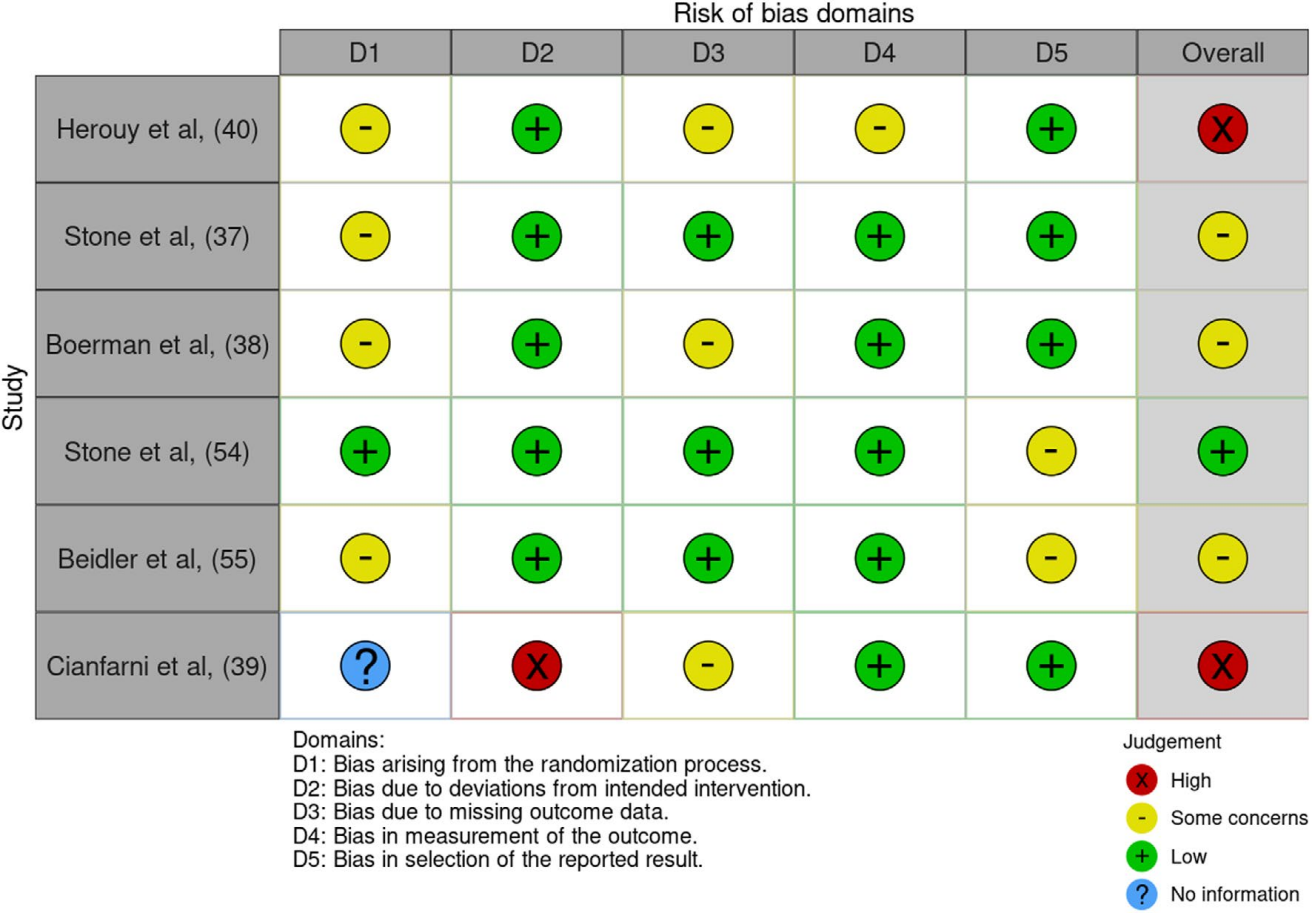
Randomised control trial (RCT) ROB2 assessment of included studies of RCTs (*n* = 5).

The largest proportion of studies took place in the United States (*n* = 12), while 21 studies across different European countries were included. There was one study each from China and Australia. The range of time the studies were undertaken was 1995–2024. Risk of bias analyses of the included studies demonstrated a range of study qualities, with several studies demonstrating poor quality due to poor reporting of methods and results depending on study type (Figures 2 and 3).

**FIGURE 4:**
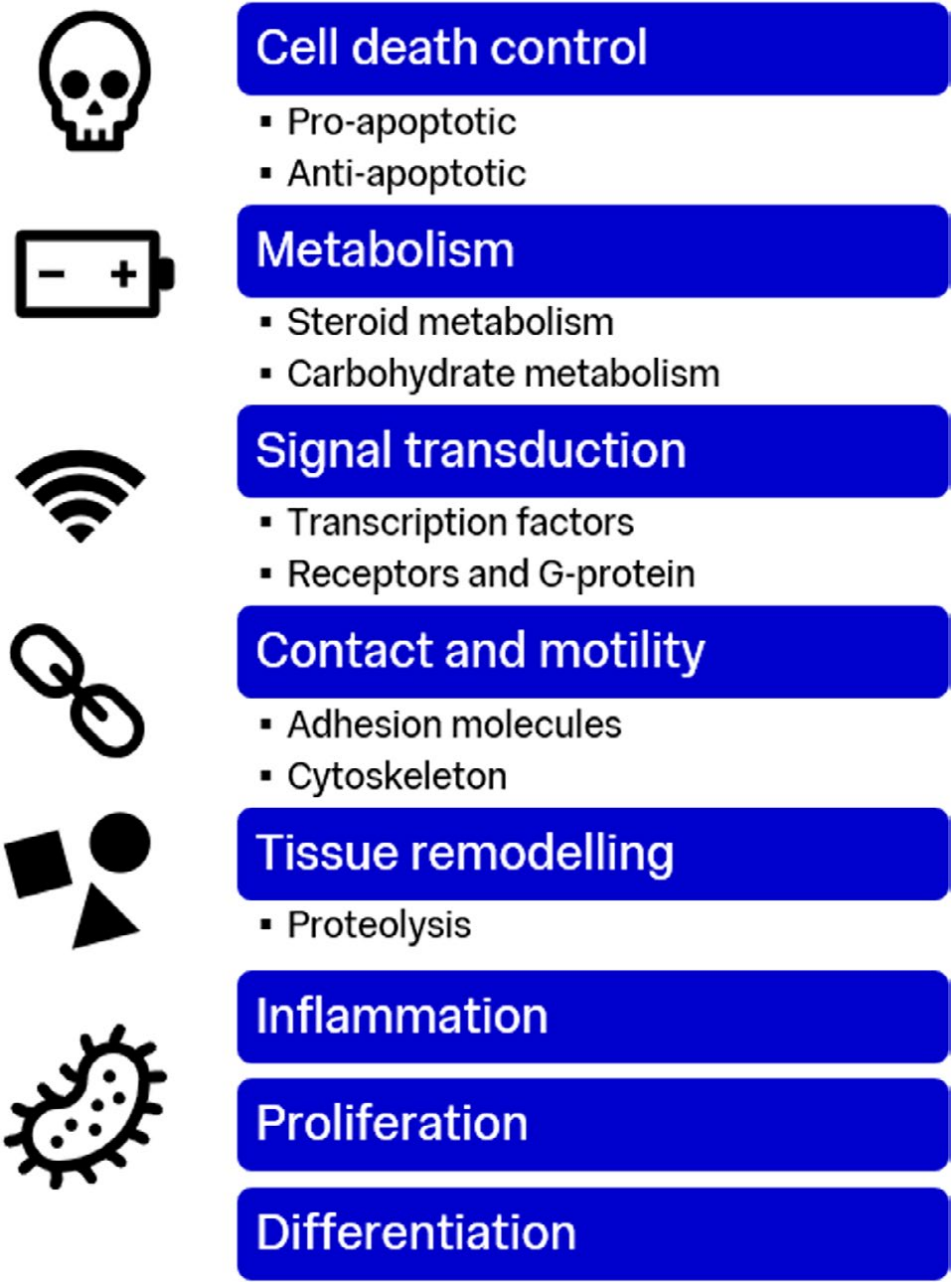
Downstream effects of RNA expression modifications.

**FIGURE 5:**
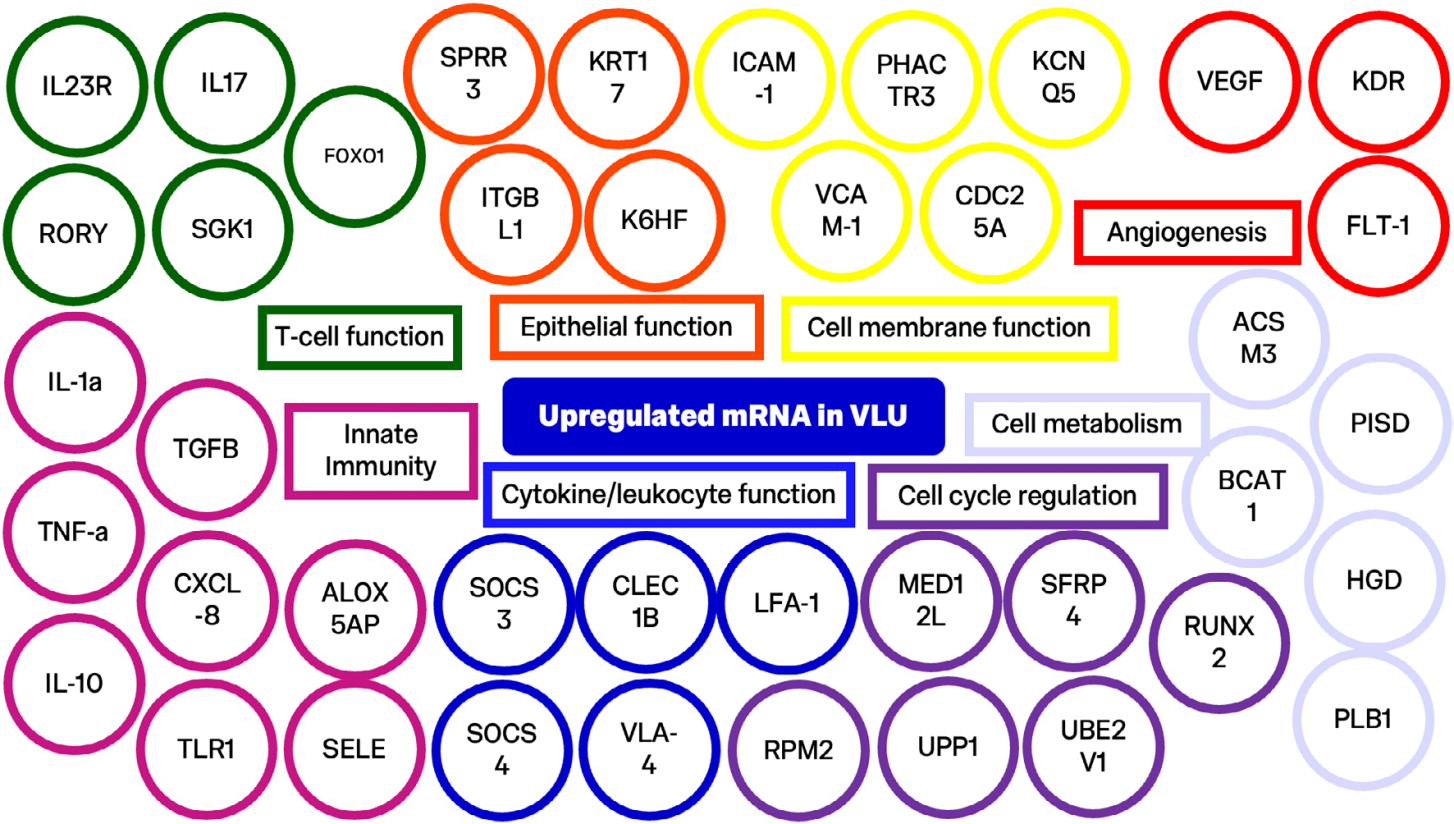
Representation of upregulated mRNAs in chronic, non‐healing VLUs and functions involved as reported in the included studies. Legend available in Data S1.

**FIGURE 6:**
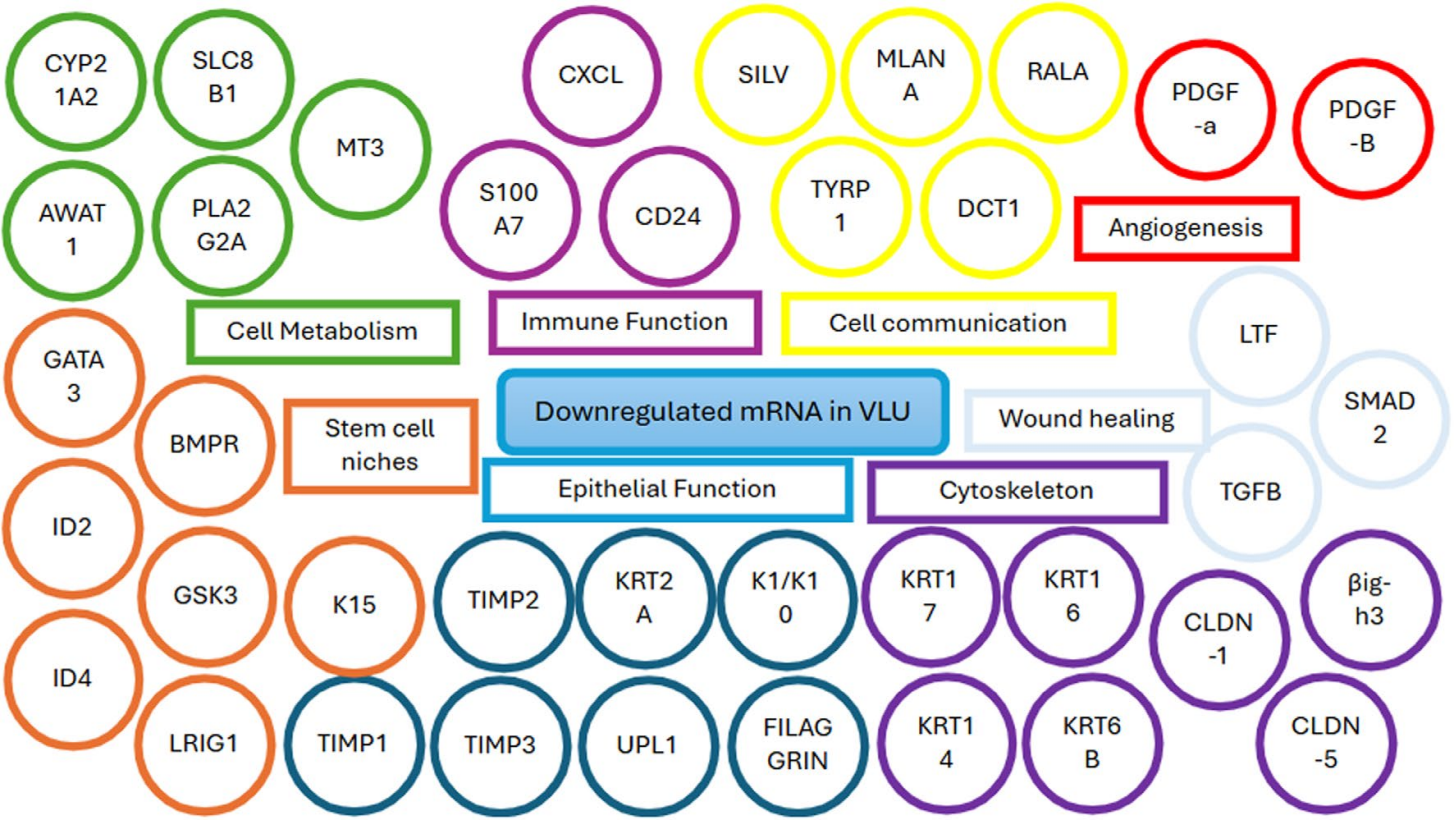
Representation of downregulated mRNA in chronic, non‐healing VLUs categorised by general function as reported in the included studies. Legend available in Data S1.

The studies were subdivided into comparisons—there were 12 studies comparing VLUs with healthy tissue from the patients themselves or other patients, seven studies comparing other wound types (e.g., arterial, psoriatic) with VLUs and five studies comparing healing VLUs versus non‐healing phenotypes of VLUs. There were seven studies reviewing interventions such as ultrasound therapy, compression therapy and bioengineered living cell constructs (BLCC) and their impact on the transcriptome. Finally, five studies described differences in microRNA or non‐coding RNA expression between VLUs and healthy skin. These comparisons allowed description of different effects of mRNA and miRNA expression on VLU phenotypes (Figures 4, 5, 6).

### 3.1 Comparisons of Healthy Tissue With VLUs

When transcriptomic profiles of skin from VLUs were compared to healthy controls from other patients or own skin, there was clear differential expression in mRNA levels (Table 3a). Patients were compared against controls with no specific matching for other clinical demographic parameters. A clinical study assessing the expression of epidermal markers in VLUs described differential regulation of 1557 genes between VLUs and healthy skin, the largest number of differences reported in this review [21]. A focus on the non‐healing edges of VLUs showed upregulation of genes such as keratin 6A, keratin 17 (K17), cyclin B1, D2 and A2, cyclin‐dependent kinase inhibitor 3, while there was downregulation of the Retinoblastoma family, vascular endothelial growth factor (VEGF), epiregulin and insulin‐like growth factor binding protein 5. These mediators were hypothesised to contribute to phenotypic differences in inflammatory states, skin structure and vasculature.

Across all studies, altered levels of mRNA transcripts suggest chronic inflammatory states, hyperproliferation and reduced wound healing. Differences were reported to exist between the different wound areas subdivided into the wound bed, non‐healing edges and surrounding epithelial skin. A comparative study of normal wounds, VLUs, vasculitis wounds and blisters described no expression of tissue inhibitor of metalloproteinases (TIMP)‐1 mRNA in the epidermis of VLUs compared to healing wounds and no expression of TIMP‐3 mRNA in epithelial cells of VLUs, and sporadic expression of TIMP‐4 protein. This dysregulation in TIMP mRNA expression affects metalloproteinases, which may lead to delayed wound healing [33].

Structural differences in VLUs compared to healthy skin reveal upregulation of small proline‐rich protein 3, K17, epithelial keratin 6hf and mRNA that contribute to the dysregulation of epithelial structures [22]. Fibroblast and keratinocyte modulation also contribute to structural defects in chronic VLUs, with a range of differential mRNA expression. Fibronectin mRNA was also upregulated in chronic VLUs with plasminogen activation [31]. The epidermal stem cell niche, which has roles in tissue homeostasis and wound healing, was dysregulated in VLUs, with bone morphogenetic protein receptor type 1A, GATA3, inhibitor of DNA binding 2 (ID2) and inhibitor of DNA binding 4 (ID4) genes downregulated in VLUs [21].

Dysregulated immunity and inflammation were hypothesised to drive development of VLUs. Cytokines and immune ligands such as interleukin‐1α (IL‐1α), tumour necrosis factor α (TNF‐α), interleukin‐10 and interleukin‐8 (CXCL‐8) mRNA expression were upregulated in VLUs, driving chronic inflammation and regulation of T‐cell immunity. Within T‐cell driven immunity, interleukin‐23R and interleukin were significantly upregulated in VLUs, representing an increase in Th17 gene profile expressions [20]. Transforming growth factor‐β (TGF‐β) pathways are attenuated in VLUs, with reduced or no expression of subtypes such as TGF‐βRII in VLUs compared with healthy skin [30, 34]. A study of different stages of venous insufficiency revealed the downstream effects of increased mRNA expression of Intercellular adhesion molecule‐1. Vascular cell adhesion molecule 1 and Lymphocyte function‐associated antigen 1/integrin α4β1, remain upregulated past stasis dermatitis to attract leukocytes, potentially resulting in tissue damage [23].

### 3.2 Other Chronic Wounds (Arterial, Psoriatic) Compared With VLUs

Comparisons between VLUs and other wound types such as arterial, psoriatic, or diabetic wounds explored similarities and differences in transcriptomic profiles between different wound pathologies (Table 3b). These highlighted the shared pathways and the key differences resulting in phenotypic characteristics of VLUs such as chronicity, hyperproliferation and non‐healing [41].

Structurally, VLUs experienced downregulation of fibroblast mRNA TGF‐β‐induced protein in comparison to healing ulcers or normal skin [30]. Matrix metalloproteinase mRNA was expressed in the basal keratinocytes of VLUs [42]. Urokinase (uPA) mRNA expression was increased in VLUs compared to decubitus ulcers [36]. Comparisons between scar tissue and VLUs revealed collagen VII, I and III mRNA expression in all samples. In comparison to psoriatic wounds, VEGF splice variant expression is increased in VLUs, suggesting increased angiogenesis [37].

Higher levels of hypoxia‐inducible factor‐1alpha protein (HIF‐1α) were detected in both the cytoplasm and the nuclei of fibroblasts and some endothelial cells in VLUs from a study investigating differences between VLUs and diabetic ulcers [39]. VLUs and diabetic ulcers shared hypoxic environments, but the lack of hyperglycaemia results in differential expression of HIF‐1α which modulates fibroblast motility and wound healing.

### 3.3 Healing Compared With Non‐Healing Phenotype of VLUs

Non‐healing phenotypes of VLUs were defined as slow healing, chronic and debilitating wounds that do not respond to conventional therapies. It should be noted, however, that these classifications varied widely across studies (Table 3c). One study defined non‐healing ulcers derived from a clinical cohort of patients with venous ulcers—wound size was measured at initial time of biopsy and at 3 months after best medical therapy, and the wounds which grew or remained static were labelled as ‘non‐healing’. In another study, non‐healing ulcers were defined as ulcers greater than 5 cm^2^ and present for longer than 6 months [43]. Finally, non‐healing ulcers were described as ulcers failing to heal in a year from entry to the study from a group of 35 patients with VLUs [44].

In non‐healing VLUs, cytokine signalling was dysregulated with TNF‐α, IL‐1α and CXCL‐8 showing increased expression with significant upregulation of TAM receptors and their ligands [25] Similarly, mRNA expression of suppressor of cytokine signalling family, VEGF189, angiopoietin‐1 and angiopoietin‐2 were found in more samples from unhealed VLUs [41].

Differential expression at wound edges of non‐healing VLUs demonstrated upregulated Secreted frizzled‐related protein 4, collagen type X alpha 1, and downregulated keratin 16, small proline‐rich protein 1B and filaggrin—however, these differences were not replicated from the cells at the wound bed between non‐healing and healing groups [43].

The development and evaluation of a ‘healing’ WounD25 and WounD14 gene signature score to predict wound outcomes from chronic VLUs comprising mRNAs such as autocrine motility factor receptor, actin‐related protein 2 and β‐catenin amongst others has been described in a series of papers from the same group [45, 46]. These demonstrated that an initial assessment of the WD14 gene signature can provide early identification of ‘healing’ and ‘non‐healing’ wound phenotypes in a series of wound biopsies, with a positive predictive value of 85.2% in 16 biopsies.

### 3.4 Trials With Interventions/Treatments

The impacts on mRNA expression of several interventions were studied in cohorts of patients with VLUs (Table 3d). Compression therapy is a mainstay of VLU management, and its impacts on the genotype include upregulation of occludin and claudin‐3 mRNA that affect tight junction function in VLU tissue.

A clinical trial demonstrated the effects of BLCCs where following BLCC treatment, downregulation of TGFβ2, tenascin C (TNC) and TIMP3 was observed. BLCC treatment induced Toll‐like receptor 4, metallothionein‐1H, metallothionein 1X and metallothionein 2A mRNA expression, alongside fibronectin‐1, TNC, secreted phosphoprotein 1, connective tissue growth factor, hepatocyte growth factor, plasminogen activator inhibitor 1 and alpha‐smooth muscle actin expression [48]. These changes induced by BLCC use encouraged remodelling of fibrotic tissue to improve healing.

Therapeutic ultrasound treatment of VLUs in a trial demonstrated upregulated phospholipase A2 group IIA, metallothionein‐3, gamma‐glutamyltransferase 3 pseudogene and exostosin like glycosyltransferase 1 mRNA compared with sham‐treated patients within 1 week of treatment [49]. These gene expression changes revealed enriched and downregulated gene sets from cell metabolism, immunity and proliferation—downregulation of inflammation was hypothesised as the mechanism for improved wound healing in ultrasound treatment. This was reflective of the healing phenotype of VLUs as described previously, where reduced expression of inflammatory factors was found.

A trial of granulocyte/macrophage colony‐stimulating factor (GM‐CSF) in non‐healing VLUs described post GM‐CSF treatment mRNA expression, with increased transcription of VEGF in PMA‐differentiated cultured monocytes (macrophages, myofibroblasts and factor XIII+ cells) after treatment [52]. Autologous platelet‐derived wound healing factor treatment also increases expression of a‐ and B‐subunits of integrin mRNA to increase angiogenetic drive in chronic wounds to improve healing [47].

### 3.5 Non‐Coding RNA Studies

Studies exploring non‐coding RNAs described their roles in regulating wound repair in VLUs. Non‐coding RNAs affect regulation of mRNA and downstream protein expression and subsequent biological pathways (Tables 2 and 3e). Paired analyses of microRNA expression demonstrate impact on RNA expression profiles, with effects on inhibition of wound healing, altering gene expression at post‐translational levels and changes in downstream reactive oxygen species generation.

**TABLE 2:** Differences in miRNA expression in VLUs.

MicroRNA expression	Mechanisms	Downstream effects
miRNA‐16, ‐20a, ‐21, ‐106a, ‐203 and –130a induced	Target 3′‐UTR of EGR3	Inhibits cutaneous wound healing
circRNAs hsa‐TNFRSF21_0001 and hsa‐CHST15_0003 upregulated		Chronic inflammation, increased extracellular matrix organisation and cell adhesion
miR‐301a‐3p induced		Endothelial cell dysfunction
miR‐34a, miR‐424 and miR‐516 upregulated		Chronic inflammatory response
WAKMAR2 downregulated	TGF‐β pathway	Chronic inflammation, keratinocyte motility

Abbreviations: circRNA, circular RNA; miRNA, micro RNA; EGR3, early growth response 3; TGF‐β, transforming growth factor‐ β; WAKMAR2, wound and keratinocyte migration‐associated long noncoding RNA 2.

**TABLE 3a:** Table of studies describing healthy tissue compared with VLUs.

Author	Citation number	Study design	cohort size	Sample type	Techniques	RNA targeted	RNA type	RNA expression
Rosario Amato et al.	[20]	Cross‐sectional clinical study	*n* = 23, including healthy control *n* = 8, venous disease (C2–C4) *n* = 8, VLUs (C6) *n* = 7	Blood, peripheral blood lymphocytes	Extraction, RT‐PCR	Th17 and genes involved in Th17 differentiation (IL23R, IL17, RORγ, TGFβ, SGK1, RANBP1 and FOXO1)	mRNA	Th17 gene expression increases in chronic venous insufficiency. IL23R and IL17 upregulated in VLUs.
Olivera Stojadinovic et al.	[21]	Cross‐sectional clinical study	*n* = 10, including VLUs *n* = 6 and healthy controls *n* = 4	Tissue	Extraction, qPCR	BMPR1a, GATA3, ID2 and ID4	mRNA	BMPR1a, GATA3, ID2 and ID4 genes downregulated in VLUs.
Harold Brem et al.	[22]	Temporal cross‐sectional study	*n* = 3, from non‐healing edge and own healthy skin	Tissue	Extraction, microarray, clustering analysis	Vimentin and perlecan (fibroblasts) and stratifin and junctional plakoglobin (keratinocytes)	mRNA	Consistent expression for perlecan, stratifin and junctional plakoglobin in all areas of wound. KRT2A, SPRL1B, Lactoferrin, TYRP1, DCT 1, SILV, MLANA, UPL1, EGFR expression suppressed in VLUs. SPRR3, KRT17, K6HF, MMP1, KLK2, IGFBP2, CEACAM6 expression induced in VLUs.
Manfred Peschen et al.	[23]	Cross‐sectional clinical study	*n* = 70, including controls *n* = 10 healthy and VLI *n* = 60 (telangiectases *n* = 14, stasis dermatitis *n* = 10, hyperpigmentation *n* = 10, lipodermatosclerosis *n* = 13, VLU *n* = 4)	Tissue	Extraction, RT‐PCR, electrophoresis	ICAM‐1, VCAM‐1, LFA‐1, VLA‐4	mRNA	High mRNA levels of ICAM‐1, VCAM‐1, LFA‐1 and VLA‐4 in VLUs.
Y. Herouy et al.	[24]	Cross‐sectional clinical study	*n* = 16, including *n* = 8 varicosis‐associated edema and *n* = 8 VLU and *n* = 8 healthy controls	Tissue	Extraction, RT‐PCR	OCLN, CLDN‐1, CLDN‐3, CLDN‐5	mRNA	OCLN and CLDN‐3 expression upregulated in chronic VLUs. After compression therapy, CLDN‐1 and CLDN‐5 mRNA expression upregulated.
Kata Filkor et al.	[25]	Cross‐sectional clinical study	*n* = 69 VLU, *n* = 42 control without VLU	Blood	Extraction, RT‐PCR	Axl, Tyro3, CXCL8, Gas6, IL‐1α, IL‐10, MerTK, ProS and TNFα	mRNA	IL‐1α, TNFα, IL‐10 and CXCL8 significantly upregulated in VLUs. TNFα, IL‐1α and CXCL‐8 showed increased expression in VLUs classified as non‐responders to treatment. TAM receptors (Tyro3, Axl, MerTK) and their ligands Gas6 and ProS expression increased in non‐responder VLU patients. Ax1 expression is upregulated and Gas6 expression downregulated in treatment responsive patients.
D. Peus et al.	[26]	Cross‐sectional clinical study	*n* = 15 VLU, with comparisons to patient's own healthy tissue	Tissue	Extraction, Northern blot	PDGF‐R	mRNA	PDGF‐R subunits mRNA were strongly expressed in VLUs.
Ivan B. Wall et al.	[27]	Cross‐sectional clinical study	*n* = 4 VLU, with matched uninvolved dermal fibroblasts	Tissue	Extraction, microarray analysis	Gene sets	mRNA	53 genes upregulated in VLUs, including MET, TUSC3, IGFBP7, etc. 65 genes demonstrated lower expression in chronic wounds, including ISLR, MAX, FYN, etc.
Olivera Stojadinovic et al.	[28]	Cross‐sectional clinical study	*n* = 3 VLU	Tissue	Extraction, microarray, clustering, QT‐PCR	Gene sets	mRNA	1557 genes differentially regulated between non‐healing edges of venous ulcers and healthy skin. K6, K17, cyclin B1, cyclin D2, cyclin A2, cyclin F, CDKN2B, CDKN3 and cyclin M4 induced at non‐healing edges of VLUs. Rb family, p107, p130, VEGF, EREG, ANGPLT6, IGFBP5, BMP‐2 and BMP‐7 were downregulated at non‐healing VLU edges.
Irena Pastar et al.	[29]	Cross‐sectional clinical study	*n* = 14, including *n* = 4 healthy and *n* = 10 VLU	Tissue	Extraction, qPCR, LOLA gene‐array data comparison	TGFβRI, TGFβRII and TGFβRIII	mRNA	TGFβ treatment suppressed 73 genes and induced 67 genes present in VLUs. TGFβRI and TGFβRIII mRNA expression reduced in nonhealing edges of VLUs compared with healthy skin. No significant changes in expression of TGFβRII, Smad2, 3 and 4 genes were found. ATF3, GADD45β and ZFP36L1 mRNA levels were downregulated in VLUs compared with healthy skin.
Allison J. Cowin et al.	[30]	Cross‐sectional clinical study	*n* = 12 VLUs, *n* = 3 healthy tissue	Tissue	Extraction, RT‐PCR, competitive RT‐PCR	TGF‐β1	mRNA	Normal expression of TGF‐B1 in VLUs. No expression of TGF‐βRII in VLUs.
Y. Herouy et al.	[31]	Cross‐sectional clinical study	*n* = 43, including *n* = 23 VLU and *n* = 20 healthy	Tissue	Extraction, RT‐PCR	uPA, tPA, uPAR, PAI‐1, PAI‐2	mRNA	uPA and uPAR mRNA expression increased in VLUs. No differences detected for tPA, PAI‐1 or PAI‐2 mRNA.
Katia C. Ongenae et al.	[32]	Cross‐sectional clinical study	*n* = 40, including *n* = 14 VLU and *n* = 11 healthy and *n* = 15 acute wounds	Tissue	Extraction, RT‐PCR, in situ hybridisation	Fibronectin	mRNA	Expression of FN mRNA Is upregulated in VLU, Integrin a5b1 is not upregulated in VLU.

*Note:* Data extraction table. Legend available in Data S1.

**TABLE 3b:** Table of studies describing alternative wounds (arterial, psoriatic) compared with VLUs.

Author	Citation number	Study design	Cohort size	Sample type	Techniques	RNA targeted	RNA Type	RNA expression
Maarit Vaalamo et al.	[33]	Cross‐sectional clinical study	*n* = 31, including VLU *n* = 14, vasculitis *n* = 5, suction ulcers *n* = 12	Tissue	In situ type hybridisation	TIMPs‐1, ‐2, ‐3 and ‐4	mRNA	TIMP‐1 not expressed in VLU epithelium. TIMP‐2 expressed in stromal fibroblast‐like cells in VLU. TIMP‐3 expressed in stromal cells in VLU, but not in epithelium.
Jisun Cha et al.	[35]	Temporal cross‐sectional study	*n* = 5, VLUs and acute wounds created on same patient	Tissue	Extraction, PCR	βig‐h3	mRNA	βig‐h3 downregulated in fibroblasts in chronic venous ulcers.
M. Weckroth et al.	[36]	Cross‐sectional clinical study	*n* = 13, including VLU *n* = 8 and decubitus ulcers *n* = 5	Tissue	Extraction, in situ hybridization	tPA, uPA, PAI‐1, uPAR and vitronectin	mRNA	tPA mRNA expression at basal dermis of ulcer margin, keratinocytes and perivascular regions. uPA mRNA expressed in 6 VLUs. PAI‐1 mRNA showed similar location in 7 VLUs.
I. Hopkinson et al.	[37].	Cross‐sectional clinical study	*n* = 6 VLU, *n* = 1 perianal excision, *n* = 11 scars	Tissue	Extraction, RT‐PCR	Collagen VII, I and III	mRNA	Collagen VII, I and III detected in all wound samples.
Gereon Lauer et al.	[38]	Cross‐sectional clinical study	*n* = 18, including *n* = 8 VLU, *n* = 6 psoriasis and *n* = 4 control	Tissue and wound fluid	Extraction, RT‐PCR, In situ hybridisation	VEGF	mRNA	VEGF splice variants (VEGF121, VEGF165 and VEGF189) expression increased in VLUs. Expression of KDR and Flt‐1 was demonstrated in VLUs.
Sergiu‐Bogdan Catrina et al.	[39]	Cross‐sectional clinical study	*n* = 5, including *n* = 2 VLU and *n* = 3 diabetic ulcers	Tissue	Extraction, RT‐PCR, reporter gene assay	HIF‐1 α	mRNA	HIF‐1α mRNA detected in both the cytoplasm and the nuclei of fibroblasts and some endothelial cells in venous ulcers.
M. Vaalamo et al.	[40]	Cross‐sectional clinical study	*n* = 14 VLU	Tissue	Extraction, in situ hybridisation	Human interstitial collagenase. Stromeiysin‐1, stromelysin‐2, uPA and TIMP‐1	mRNA	Collagenase mRNA detected in basal keratinocytes of VLU. Stromelysin‐1 mRNA expressed in nine of 14 chronic ulcers adjacent but distal to wound edge. Stromelysin‐2 mRNA detected in basal keratinocytes of the wound edge tip. uPa mRNA expressed in 10 of 12 chronic ulcers at wound edge. TIMP‐1 never detected in epidermis of chronic wounds. Matrilysin not expressed.

*Note:* Data extraction table. Legend available in Data S1.

**TABLE 3c:** Table of studies describing healing compared with non‐healing phenotype of VLUs.

Study details		Method						Results
Author	Citation number	Study design	Cohort size	Sample type	Techniques	RNA targeted	RNA type	RNA expression
Yi Feng et al.	[41]	Temporal cross‐sectional study	*n* = 71, including ‘healing/healed’ *n* = 20 ‘non‐healing’ VLUs *n* = 51	Tissue	Extraction, RT‐PCR	SOCS family, cytokeratin 19 gene	cRNA	SOCS3 and SOCS4 transcription upregulated in non‐healing chronic wound tissue. Non‐significant increase in expression of SOCS1, 2, 5 and 6 in non‐healing VLUs.
Carlos A. Charles et al.	[43]	Cross‐sectional clinical study	*n* = 10, including *n* = 5 nonhealing and *n* = 5 healing chronic VLU	Tissue	Extraction, microarray, image analysis	Gene sets	mRNA	Secreted frizzled‐related protein 4 and collagen type X alpha 1 upregulated in non‐healing wound edges of VLUs. Keratin 16, small proline‐rich protein 1B, and filaggrin downregulated from non‐healing wound edges of VLUs. From cells at wound bed, less differences in mRNA expression between non‐healing and healing groups.
Susan L. Drinkwater et al.	[44]	Cross‐sectional clinical study	*n* = 32, including *n* = 19 healing and *n* = 13 nonhealing VLU	Tissue and wound fluid	Extraction, RT‐PCR	VEGF splices (VEGF121, VEGF189, VEGF165 and VEGF‐R1/2; ang‐1 and ang‐2 and receptor, Tie‐2)	mRNA	VEGF121 mRNA expressed in all samples, and VEGF165 mRNA was found in 43/48 samples. mRNA expression of VEGF189, Ang‐1 and Ang‐2 were found in more samples from unhealed VLUs.
David C. Bosanquet et al. [45]	[45]	Longitudinal cohort study	*n* = 85, with *n* = 16 patients with sequential biopsies	Tissue	Extraction, RT‐PCR	WD14 gene signature	mRNA	WD14 gene signature expressed in wounds which were identified as difficult to heal

*Note:* Data extraction table. Legend available in Data S1.

**TABLE 3d:** Table of studies describing randomised control trials.

Author	Citation number	Study design	Cohort size	Sample type	Techniques	RNA targeted	RNA type	RNA expression
Y. Herouy et al.	[47]	Randomised pilot study	*n* = 23, including *n* = 12 undergoing autologous PDWHF and placebo *n* = 11	Blood and tissue	Extraction, RT‐PCR	a‐ and B‐subunits	mRNA	mRNA of a‐ and B‐subunits of integrin upregulated after autologous PDWHF treatment.
Rivka C. Stone et al.	[48]	Randomised clinical trial	*n* = 24, including *n* = 15 treatment with bilayered living cellular construct (BLCC) five weekly, and *n* = 9 control compression dressings	Tissue	Extraction, gene expression microarrays, gene ontology	TGFβ, hepatocyte growth factor (HGF), vascular endothelial growth factor (VEGF), platelet‐derived growth factor (PDGF)	mRNA	CASP14 expression upregulated in chronic VLUs vs. BLCC treatment cohort. ITM2A expression downregulated and DIO2 upregulated in BLCC treatment cohort. BLCC treatment induced Toll‐like receptor 4 expression.
Olivia Boerman et al.	[49]	Randomised clinical trial	*n* = 32, of these *n* = 7 analysed (*n* = 3 ultrasound treated and *n* = 4 sham treated)	Tissue	Extraction, cDNA library preparation, templating, enrichment and sequencing	Gene sets	mRNA	Upregulated genes in VLUs treated with ultrasound were PLA2G2A, MT3, GGT3P, EXTL1, etc. Downregulated genes in VLU treated with ultrasound were PHACTR3, HGD, MED12L, GP9, etc. Inflammatory response gene set was significantly downregulated in the ultrasound‐treated patients compared to sham‐treated patients 1 week after treatment.
Rivka Stone et al.	[50]	Randomised clinical trial	*n* = 19, including *n* = 8 control and *n* = 11 BLCC applications	Tissue	Extraction, microarray analysis, acute dermal profiles, QPCR validation, pathway analysis	Gene sets	mRNA	FN1, TNC, SPP1, CTGF, HGF, PAI‐1 and α‐SMA expression upregulated in patients with VLUs. Following BLCC treatment, TGFB2, tenascin C, TIMP3 expression were downregulated. BLCC treatment induced expression of MT1H, MT1X and MT2A.
Stephanie K. Beidler et al.	[51]	Cross‐sectional clinical study	*n* = 37, including *n* = 8 healthy and *n* = 14 pre‐ and *n* = 15 posttreatment	Tissue	Extraction, RT‐PCR	MMP1, 2, 3, 8, 9, as well as TIMP1 and 2	mRNA	After therapy, MMP1, 3 and 9 upregulated in VLUs.
F. Cianfarni et al.	[52]	Clinical study	*n* = 8	Tissue	Extraction, in situ hybridisation, RNA isolation and Northern blot analysis	VEGF and PIGF	mRNA	After treatment with GM‐CSF, VEGF transcription increased in differentiated cultured monocytes. Cells transcribing VEGF were macrophages, myofibroblasts and factor XIII+ cells.

*Note:* Data extraction table. Legend available in Data S1.

**TABLE 3e:** Table of studies describing non‐coding RNA studies.

Study details		Method						Results
Author	Citation number	Study design	Cohort size	Sample type	Techniques	RNA targeted	RNA type	RNA expression
Maria A. Toma et al.	[53]	Cross‐sectional clinical study	*n* = 22, including control *n* = 10 and VLU *n* = 12	Tissue	Extraction, RT‐PCR, DNA electrophoresis and Sanger sequencing. Co‐expression network analysis. Gene expression microarray, gene set enrichment analysis	circRNA	circRNA	VLUs express 149 circRNAs in M7, and 68 circRNAs in M10. circRNAs hsa‐TNFRSF21_0001 and hsa‐CHST15_0003 upregulated in the keratinocytes of VLUs. M9, M15 and M14 expression upregulate din VLUs. hsa‐CHST15_0003 and SH2B3 expression were upregulated in VLUs. miR‐125b‐5p and let‐7a levels were lower in VLUs.
Irena Pastar et al.	[54]	Cross‐sectional clinical study	*n* = 15, including *n* = 10 VLU and *n* = 5 control	Tissue	Extraction, RT‐PCR	miRNA‐16, ‐20a, ‐21, ‐106a, ‐203 and ‐130a	microRNA	miRNA‐16, ‐20a, ‐21, ‐106a, ‐203 and ‐130a are induced in VLUs. Five miRNAs found to be induced in VUs can target 3′‐UTR of EGR3.
Ying Wang et al.	[55]	Cross‐sectional clinical study	*n* = 45, including *n* = 15 control, *n* = 15 C1–C3, *n* = 15 C4–C6	Blood	Extraction, RT‐qPCR	IGF1, MMP1, MMP3, MMP9, IL‐1β and TNFα	microRNA	VLU patients had increased miR‐301a‐3p, MMP1, MMP3, MMP9, IL‐1β and TNFα expression and decline in IGF1 expression in their serum.
Zhuang Liu et al.	[56]	Cross‐sectional clinical study	*n* = 22, including *n* = 10 healthy and *n* = 12 VLU	Tissue	Extraction, DE analysis, gene ontology, integrative analysis, expression correlation and target enrichment	Gene sets and miRNA sets	microRNA, mRNA	17 miRNAs identified in VLU, including miR‐34a, miR‐424 and miR‐516 which were upregulated. Downregulated M1, M3, M5, M7 and upregulated M9 in VLUs.
Eva K. Herter et al.	[57]	Cross‐sectional clinical study	*n* = 13 VLU	Tissue	Extraction, RT‐PCR	WAKMAR2	lncRNA	WAKMAR2 expression is downregulated in wound‐edge keratinocytes of human chronic wounds, i.e., VLUs. WAKMAR2 expression is induced by TGF‐β signalling in keratinocytes.

*Note:* Data extraction table. Legend available in Data S1.

Studies with correlation network analysis between circRNAs and mRNA investigating the regulatory mechanisms of circRNAs hsa‐CHST15_0003 and hsa‐TNFRSF21_0001 showed the restriction of keratinocyte migration in the epithelium but increase keratinocyte proliferation in VLUs [53].

Conversely, the inhibition of proliferation of keratinocytes was identified in a microRNA study of VLUs, where miR‐34a, miR‐424 and miR‐516 upregulation resulted in promotion of an inflammatory response [56]. Regulation of endothelial cell damage in VLUs is also affected by the presence of miR‐301a‐3p which was overexpressed in patients with VLUs [55]. Finally, wound and keratinocyte migration‐associated long noncoding RNA 2 affected keratinocyte production of inflammatory mediators, regulation of wound healing and wound chronicity through its effects as an RNA polymerase transcript [57].

### 3.6 Summary of Results

The transcriptomic profile of VLU tissue compared with healthy tissue reflects a state of chronic inflammation, dysregulated immunity and vascular dysfunction. Severe non‐healing VLUs show differential expression of mRNAs related to immunity and vasculature. In VLUs, non‐coding RNA impacts mRNA expression with knock‐on effects on keratinocyte and endothelial cell function. While compared with other wound aetiologies, shared pathways in fibroblast dysregulation and scar formation are highlighted as key drivers of ulcer formation; while diabetic wounds highlight the impact of hyperglycaemia impacting wound healing. Finally, therapeutic interventions such as ultrasound therapy and BLCCs on VLUs stimulate healing and tissue remodelling in patient cohorts.

## 4 Discussion

This systematic review has provided a comprehensive summary of studies reporting transcriptomic data in patients with VLUs across a 20‐year period. Transcriptomic analysis of VLUs aims to improve the comprehension of the underlying biology of VLUs by identifying key RNA transcripts that drive this pathological manifestation. Increased understanding of the role of non‐coding RNAs such as miRNAs and the regulation of transcription may also be useful in delineating synthetic and regulatory pathways that govern VLU pathophysiology.

Acute wound healing relies on four main stages—haemostasis, inflammation, proliferation and remodelling, which is modulated through a series of complex genetic, mRNA, ncRNA and downstream interactions [58, 59]. Chronicity in VLUs may be driven by both prolonged inflammation and poor remodelling, highlighted by the various transcriptomic products that have been investigated in the studies included in this review.

Distinct gene signatures with transcription molecules involved in chronic inflammation, immune signalling and wound healing have been identified through the literature. Patients with VLUs were more likely to suffer from increased chronic inflammation and decreased wound healing, with variable molecular expression depending on the location of biopsies. Different wound subregions demonstrate distinct transcriptomic differences in mRNA expression—wound edges suppress keratinocyte differentiation through downregulation of K1 and K10 resulting in over‐epithelisation and granulation of the edges [34].

Fundamental differences have been described between systemic and local inflammation, where VLUs express chronic ongoing inflammation that can cause dysregulation of signalling molecules. Intrinsic differences have been observed between healers and non‐healers in VLUs, where non‐healers expressed dysregulation in extracellular matrix RNA and structural protein RNA expression leading to chronicity of wounds. The healing versus non‐healing phenotypes of VLUs may also be attributed to persistence of chronic inflammation impairing wound healing—low‐frequency and low‐intensity ultrasound treatment has shown downregulation of inflammatory gene sets improving healing [49]. These changes in immunity and angiogenesis reveal that non‐healing ulcers express higher levels of angiogenetic factors and chronic inflammatory molecules that drive their chronicity.

Supportive evidence of therapies such as ultrasound and BLCC in randomised controlled trials have been described in the literature. Nevertheless, studies describe changes in mediators with treatment (ultrasound, GM‐CSF), which implies involvement in biological processes driving healing. The use of BLCC to trigger acute inflammation to override chronic inflammation of non‐healing VLUs via diminishing the WNT‐B‐catenin pathway and activation of keratinocyte mRNA has been described with great clinical efficacy [48]. The identification of such is foundational for development of future therapies, providing preliminary data to drive future developments and maximise wound healing. However, these studies are limited by small sample sizes and short follow‐up periods. There is further work to be done before large‐scale translation to clinical practice [58].

ncRNAs are an important regulator of mRNA expression, modulating gene expression at a pre‐ and post‐translational level. Analyses of microRNA expression in the studies demonstrate a wide‐ranging impact, altering gene expression related to wound healing and reactive oxygen species generation. For instance, through knockdown studies, the circRNAs hsa‐TNFRSF21_0001 and hsa‐CHST15_0003 found to be upregulated in VLUs have downstream effects on expression of the TIMP1, VEGF, E2F1 and CCNE1, affecting cell proliferation and migration of keratinocytes. Similarly, over‐expression of the miR‐301a‐3p ncRNA increases oxidative stress and pro‐inflammatory pathways through targeting IGF1 and downstream pathways [55]. Further clinical studies identified pathologically expressed miRNAs including miR‐34a, miR‐424 and miR‐516, which suppressed keratinocyte migration and proliferation through complex co‐operative networks affecting various miRNAs and mRNAs [56]. VLUs demonstrate abnormal circRNA expression patterns which have also been reported in diabetic foot wounds [53, 60, 61]. Further research is required to delineate the complexity of the interactions of ncRNAs with their targets and functions in the VLU healing pathways.

Due to the high heterogeneity of studies and the research methods, a meta‐analysis was not performed. There was also poor reporting of specific study details including wound sizes, chronicity and no control matching. Studies also do not consistently report on downstream protein expression. This limits the power of our study. Each study reports different results and sets varied significance levels, which limits generalisability. Patients described in the studies were heterogenous populations without control of other co‐morbidities such as diabetes or peripheral arterial disease, which causes differences to be attributable to other diseases and not VLUs alone. The transcriptomics of VLUs have not been clearly studied in different ethnicities, which may impact the generalisability of research findings to global practice. Finally, as most studies conducted are primary in nature, it is difficult to draw direct clinical correlations and applicability to practice.

Further research must be conducted to integrate a multi‐omics perspective including genomic, transcriptomic, proteomic and metabolomic analysis. As non‐coding RNA are differentially expressed, there may be multiple regulatory steps that an isolated ‐omics technique will be unable to identify. Full understanding of the relationships of upstream genetics and downstream proteomics can capture important data to paint the full picture driving the development and chronicity of VLUs. With the advent of transcriptomic technology, studies can focus upon NGS which has greater sensitivity to amplify relevant genes. The potential for topical or systemic therapies to upregulate ‘healing’ pathways and downregulate chronic inflammation are yet to be explored. Development of clinically relevant diagnostic and management tools requires further proof‐of‐concept and large‐scale research studies to demonstrate safety and efficacy in future.

## 5 Conclusion

This systematic review demonstrates that the clinical manifestations of VLUs are associated with transcriptomic differences in VLUs. These underlying biological pathways in the pathogenesis of VLUs are clearly complex. There is much to learn about the role of non‐coding RNA that can modulate the transcriptome to affect phenotypic representation. Further studies integrating all steps of the pathway through multi‐omics can aid in understanding the clinical phenotype of patients.

## Author Contributions

C.L.S. and K.H.M.T. conceptualised, conducted abstract and full‐text screening, extraction and write‐up. A.H.D. and S.O. conducted the review of the paper as senior authors.

## Funding

The authors have nothing to report.

## Ethics Statement

The authors have nothing to report.

## Conflicts of Interest

The authors declare no conflicts of interest.

## Supporting information


**Data S1:** wrr70140‐sup‐0001‐Supinfo.docx.

## Data Availability

Data sharing not applicable to this article as no datasets were generated or analysed during the current study.
