# Supplementary material for: Transcriptomics in Venous Leg Ulcers (VLU): A Systematic Review
Source: Wound Repair Regen. 2026 Mar 5;34(2):e70140. doi: 10.1111/wrr.70140 (PMC12963810; doi:10.1111/wrr.70140)
Supplement: Supplementary file 1 — Data S1: wrr70140‐sup‐0001‐Supinfo.docx. [file WRR-34-0-s001.docx]

Supplemental Material – Legends for Figures and Tables

Table 3a: Table of studies describing healthy tissue compared with VLUs

Legend:

VLU: Venous leg ulcer

RT-PCR: Reverse transcriptase polymerase chain reaction

qPCR: Quantitative polymerase chain reaction

IL23R: Interleukin-23 receptor

IL17: Interleukin-17

RORγ: RAR-related orphan receptor gamma

TGFβ: Transforming growth factor beta

SGK1: Serum and glucocorticoid-regulated kinase 1

RANBP1: Ran-specific binding protein 1

FOXO1: forkhead box O1

BMPR1a: Bone Morphogenetic Protein Receptor, type 1A

ID2: Inhibitor of DNA binding 2

ID4: Inhibitor of DNA binding 4

KRT2A: Keratin 2

SPRL1B: Small proline-rich protein-1B

TYRP1: Tyrosinase-related protein 1

DCT 1: dopachrome tautomerase

SILV: Silver homologue

MLANA: Melan-A

UPL1: ubiquitin-protein ligase 1

EGFR: epidermal growth factor

SPRR3: Small Proline-Rich Protein 3

KRT17: Keratin 17

K6HF: Type II epithelial keratin 6hf

MMP1: matrix metallopeptidase 1

KLK2: kallikrein related peptidase 2

IGFBP2: Insulin-like Growth Factor Binding Protein 2

CEACAM6: Carcinoembryonic Antigen-related Cell Adhesion Molecule 6

ICAM-1: Intercellular Adhesion Molecule 1

VCAM-1: Vascular Cell Adhesion Molecule 1

LFA-1: Lymphocyte function-associated antigen 1

VLA-4: Integrin α4β1

OCLN: Occludin

CLDN: Claudin

Tyro3: TYRO3 protein tyrosine kinase

CXCL8: Chemokine ligand 8

Gas6: Growth arrest specific 6

IL-1α: Interleukin 1-alpha

IL-10: Interleukin 10

MerTK: Mer tyrosine kinase receptor

ProS : Protein S

TNFα: Tumor Necrosis Factor alpha

PDGF-R: platelet-derived growth factor receptor

MET: Hepatocyte growth factor receptor

TUSC3: tumor suppressor candidate 3

IGFBP7: Insulin-like Growth Factor-binding Protein 7

ISLR: immunoglobulin superfamily containing leucine rich repeat

MAX: MYC associated factor X

K6: Keratin 6

K17: Keratin 17

CDKN2B: cyclin dependent kinase inhibitor 2B

CDKN3: cyclin dependent kinase inhibitor 3

Rb: Retinoblastoma

VEGF: vascular endothelial growth factor

EREG: Epiregulin

ANGPLT6: Angiopoietin like 6

IGFBP5: Insulin like binding growth factor 5

BMP-2: bone morphogenetic protein 2

BMP-7: bone morphogenetic protein 7

ATF3: activating transcription factor 3

GADD45β: growth arrest and DNA damage inducible beta

ZFP36L1: ZFP36 ring finger protein like 1

uPA: plasminogen activator, urokinase

tPA: plasminogen activator, tissue type

uPAR: plasminogen activator, urokinase receptor

PAI-1: Plasminogen Activator Inhibitor-1

PAI-2: Plasminogen Activator Inhibitor-2

Table 3b: Table of studies describing alternative wounds (arterial, psoriatic) compared with VLUs

Legend:

VLU: Venous leg ulcer

RT-PCR: Reverse transcriptase polymerase chain reaction

TIMP: tissue inhibitor of metalloproteinases

βig-h3: Transforming Growth Factor–β–Inducible Gene

uPA: plasminogen activator, urokinase

tPA: plasminogen activator, tissue type

uPAR: plasminogen activator, urokinase receptor

PAI-1: Plasminogen Activator Inhibitor-1

VEGF: vascular endothelial growth factor

HIF-1α: Hypoxia-inducible factor-1α

Table 3c: Table of studies describing healing compared with non-healing phenotype of VLUs

Legend:

VLU: Venous leg ulcer

RT-PCR: Reverse transcriptase polymerase chain reaction

SOCS: Suppressor of Cytokine Signaling

VEGF: vascular endothelial growth factor

Ang-1: Angiopoietin 1

Ang-2: Angiopoietin 2

WD14: WouND 14

Table 3d: Table of studies describing randomised control trials

Legend:

VLU: Venous leg ulcer

RT-PCR: Reverse transcriptase polymerase chain reaction

RNA: ribonucleic acid

mRNA: Messenger ribonucleic acid

PDWHF: platelet-derived wound healing factors

BLCC: bilayered living cellular construct

TGFβ: transforming growth factor beta

HGF: hepatocyte growth factor

VEGF: vascular endothelial growth factor

PDGF: platelet-derived growth factor

MMP1: matrix metallopeptidase 1

TIMP: Tissue inhibitors of metalloproteinases

PIGF: Placenta Growth Factor

ITM2A: Integral membrane protein 2A

PLA2G2A: phospholipase A2 group IIA

MT3: metallothionein 3

GGT3P: gamma-glutamyltransferase 3 pseudogene

EXTL1: Exostosin Like Glycosyltransferase 1

PHACTR3: Phosphatase and actin regulator 3

HGD: homogentisate 1,2 dioxygenase

MED12L: Mediator complex subunit 12-like

GP9: Glycoprotein IX Platelet

FN1: fibronectin 1

TNC: Tenascin-C

SPP1: secreted phosphoprotein 1

CTGF: Connective Tissue Growth Factor

PAI-1: Plasminogen Activator Inhibitor-1

α-SMA: alpha-smooth muscle actin

TGFB2: Transforming Growth Factor beta 2

MT1H: Metallothionein-1H

MT1X: Metallothionein 1X

MT2A: metallothionein 2A

GM-CSF: Granulocyte-Macrophage Colony-Stimulating Factor

Table 3e: Table of studies describing non-coding RNA studies

Legend:

VLU: Venous leg ulcer

RT-PCR: Reverse transcriptase polymerase chain reaction

DNA: Deoxyribonucleic acid

CircRNA: Circular ribonucleic acid

EGR3: early growth response 3

TNFα: Tumor Necrosis Factor alpha

IGF1: Insulin-like Growth Factor 1

MMP: Matrix Metalloproteinases

IL-1β: Interleukin-1 beta

TNFα: Tumor Necrosis Factor alpha

WARKMAR2: wound and keratinocyte migration-associated long noncoding RNA 2

Figure 2: Representation of upregulated mRNAs in chronic, non-healing VLUs and functions involved as reported in the included studies.

Legend:

IL23R: Interleukin-23 receptor

IL17: Interleukin-17

RORγ: RAR-related orphan receptor gamma

SGK1: Serum and glucocorticoid-regulated kinase 1

FOXO1: forkhead box O1

SPRR3: Small Proline-Rich Protein 3

KRT1: Keratin 1

ITGBL1: integrin subunit beta like 1

K6HF: Type II epithelial keratin 6hf

ICAM-1: Intercellular Adhesion Molecule 1

VCAM-1: Vascular Cell Adhesion Molecule 1

PHACTR3: Phosphatase and actin regulator 3

CDC25A: Cell division cycle 25 phosphatase

KCNQ5: potassium voltage-gated channel subfamily Q member 5

VEGF: vascular endothelial growth factor

KDR: Kinase Insert Domain Receptor

FLT-1: Fms Related Receptor Tyrosine Kinase 1

ACSM3: Acyl-CoA Synthetase Medium Chain Family Member 3

PISD: phosphatidylserine decarboxylase

BCAT1: branched-chain amino acid transaminase 1

HGD: homogentisate 1,2 dioxygenase

PLB1: Phospholipase B1

IL-1a: Interleukin-1 alpha

TNFa: tumor necrosis factor alpha

IL-10: Interleukin-10

TGFB: transforming growth factor beta

CXCL-8: Chemokine ligand 8

TLR1: Toll Like Receptor 1

ALOX5AP: arachidonate 5-lipoxygenase activating protein

SELE: E-selectin

SOCS3: Suppressor of Cytokine Signaling 3

SOCS4: Suppressor of Cytokine Signaling 4

VLA-4: very late antigen-4

CLEC1B: C-type lectin-like receptor

LFA-1: Lymphocyte function-associated antigen 1

RPM2: Ribonuclease P protein component

MED12L: Mediator complex subunit 12-like

UPP1: uridine phosphorylase 1

SFRP4: secreted frizzled-related protein 4

UBE2V1: Ubiquitin Conjugating Enzyme E2 V1

RUNX2: RUNX family transcription factor 2

Figure 3: Representation of downregulated mRNA in chronic, non-healing VLUs categorised by general function as reported in the included studies. Legend available in supplementary file.

Legend:

CYP21A2: cytochrome P450 family 21 subfamily A member 2

SLC8B1: solute carrier family 8 member B1

MT3: metallothionein 3

PLA2G2A: phospholipase A2 group IIA

AWAT1: acyl-CoA wax alcohol acyltransferase 1

GATA3: GATA binding protein 3

ID2: inhibitor of DNA binding 2

ID4: inhibitor of DNA binding 4

GSK3: Glycogen synthase kinase-3

BMPR: bone morphogenetic protein receptor type 2

LRIG1: leucine rich repeats and immunoglobulin like domains 1

K15: Keratin 15

CXCL: Chemokine ligand

S100A7: S100 calcium binding protein A7

CD24: CD24 molecule

TIMP1: tissue inhibitor of metalloproteinase 1

TIMP2: tissue inhibitor of metalloproteinase 2

TIMP3: tissue inhibitor of metalloproteinase 3

KRT2A: Keratin 2A

UPL1: E3 ubiquitin ligase-like protein

K1: Keratin 1

SILV: Silver homologue

MLANA: Melan-A

RALA: RAS like proto-oncogene A

TYRP1: tyrosinase-related protein 1

DCT1: divalent metal transporter 1

PDGF-a: platelet-derived growth factor alpha

PDGF-B: platelet-derived growth factor beta

LTF: Lactotransferrin

SMAD2: SMAD family member 2

TGFB: transforming growth factor beta

KRT1: Keratin 1

KRT16: Keratin 16

KRT14: Keratin 14

KRT6B: Keratin 6B

CLDN-1: Claudin-1

CLDN-5: Claudin-5

Big-H3: TGFβ-Induced Gene Human Clone 3
